# Supplementary material for: Adolescent alcohol consumption produces long term changes in response inhibition and orbitofrontal-striatal activity in a sex-specific manner
Source: Dev Cogn Neurosci. 2025 Mar 19;73:101552. doi: 10.1016/j.dcn.2025.101552 (PMC11984599; doi:10.1016/j.dcn.2025.101552)
Supplement: Supplementary file 1 — Supplementary material [file mmc1.docx]

**Supplemental Figures**

**
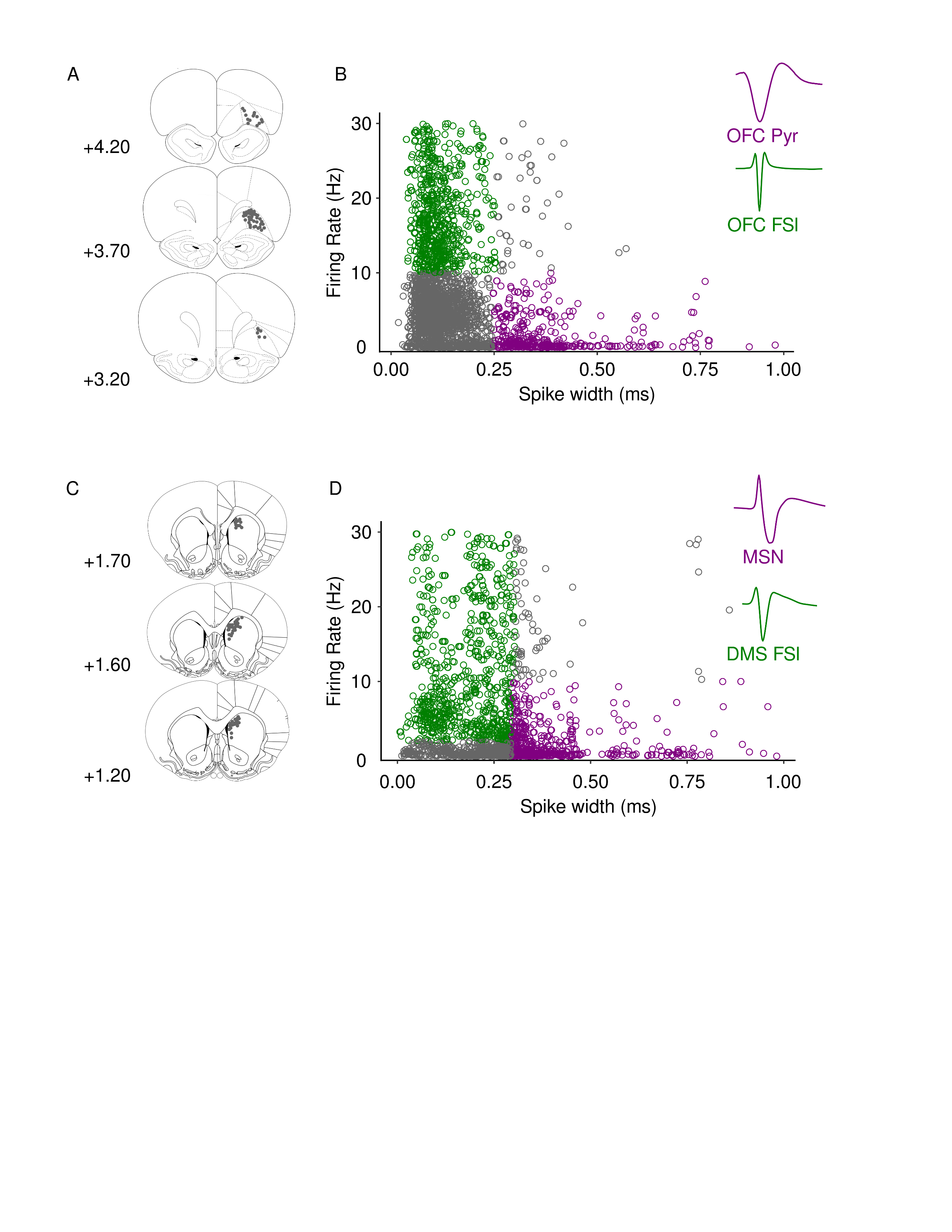
**

**Supplemental Figure 1. Histology and cell classification.**

(A) Histological placement of OFC probes. (B) Classification of OFC Pyramidal cells (Pyr; purple), fast spiking interneurons (FSI; green), and representative waveforms. (C) Histological placement of DMS probes. (D) Classification of DMS medium spiny neurons (MSN; purple) and fast spiking interneurons (FSI; green), and representative waveforms.


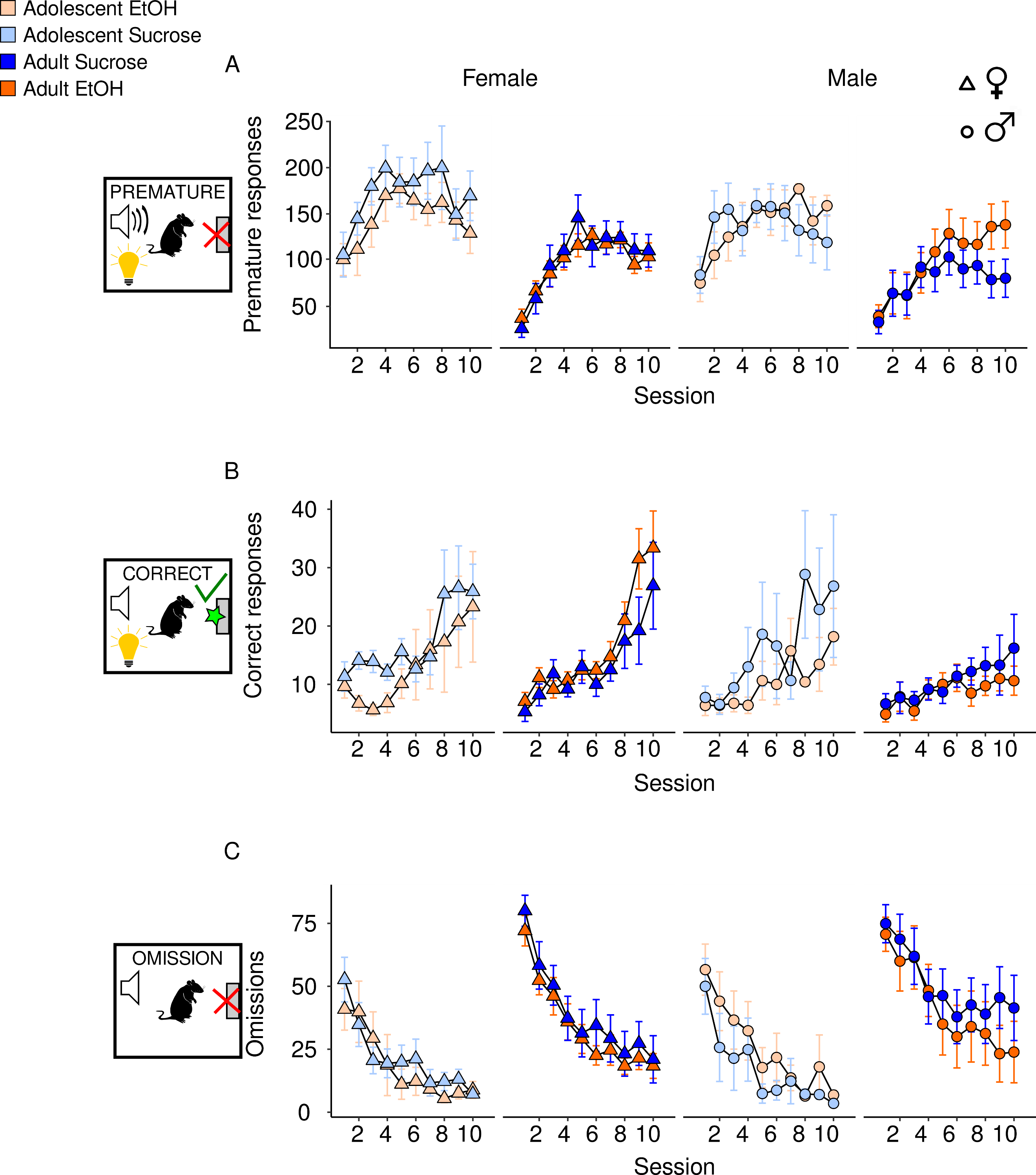


**Supplemental Figure 2. Session by Session CRIT behavioral performance.** Behavior in CRIT can be segregated by three distinct trial types: (A) Premature, (B) Correct, and (C) omission. Male (circle) and female (triangle) rats who drank EtOH (orange) or sucrose (blue) performed ten days of CRIT. Lighter shades of each color reflect adolescents and darker shades reflect adults. Bar graphs depict behavioral performance for each group aggregated across the last four sessions of CRIT. Both the number of premature (A) and correct (B) responses increased over session, while the number of omissions decreased for both males and females (C). Data are presented as mean + SEM.


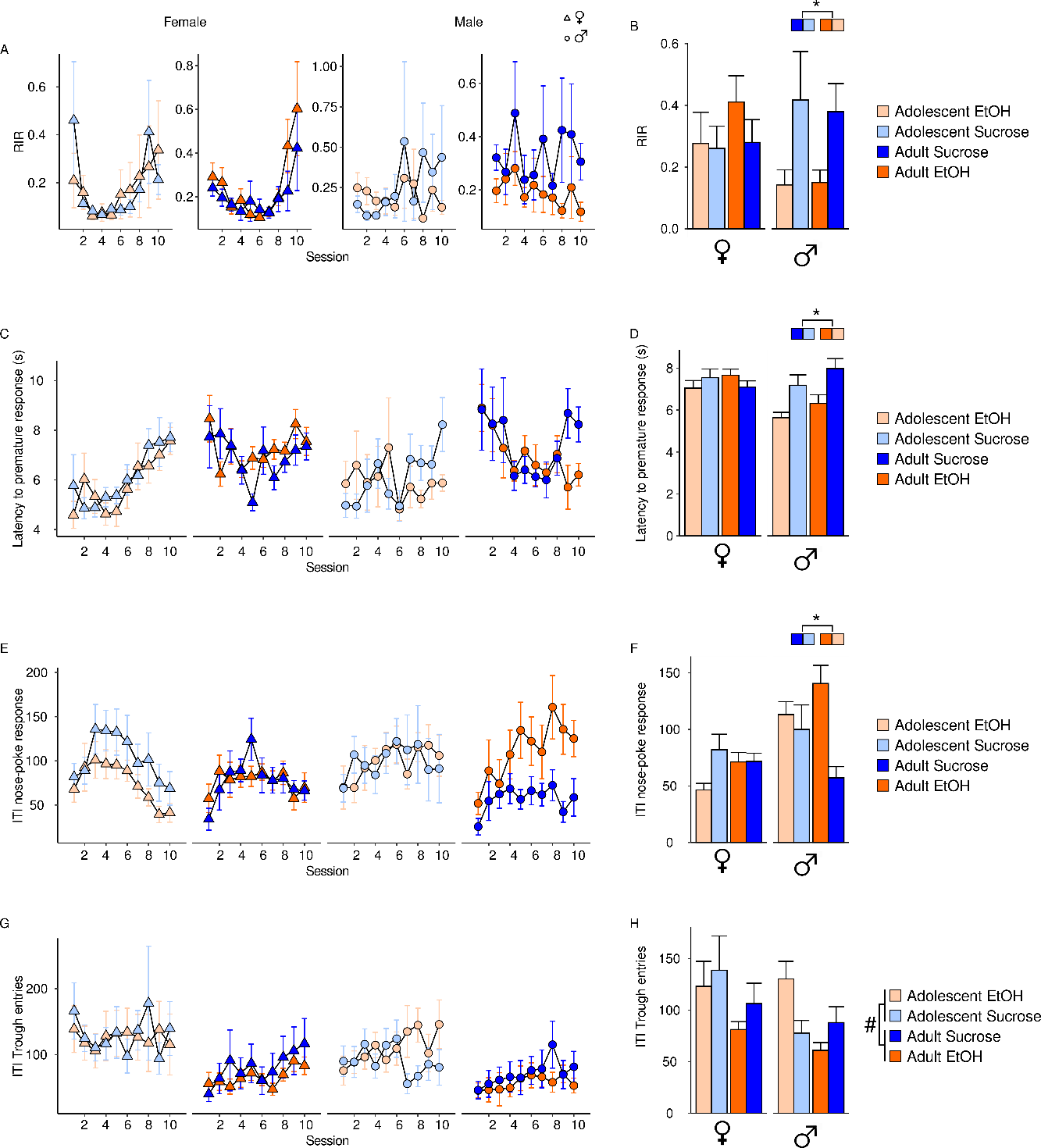


**Supplemental Figure 3. CRIT Behavior.** Behavioral measures across session (A,C,E,G) and aggregated across last 4 sessions (B,D,F,H) in female (triangle) and male (circle) rats who consumed EtOH (orange) or sucrose (blue). Adolescents are represented by the lighter shade of each color. (A) Response inhibition ratio (RIR) increased across session in females but not males. (B) RIR over the last four CRIT sessions was reduced in males who drank EtOH in adolescence, but not females. (C) Latency to make a premature response changed across sessions in adolescent and adult females and adult males. (D) Adolescent EtOH exposure was associated with reduced latencies to make a premature response in males but not females. (E) Number of nose-poke responses during the ITI changed across session in females but not males. (F) Adolescent alcohol drinking is associated with increased responding during the ITI in males but not females. (G) Number of trough entries during the ITI changed in female adults only. (H) Adolescents made more ITI trough entries than adults. Data are presented as mean + SEM. †p<0.05, main effect of sex, #p<0.05 main effect of age, *p<0.05 main effect of group.

**
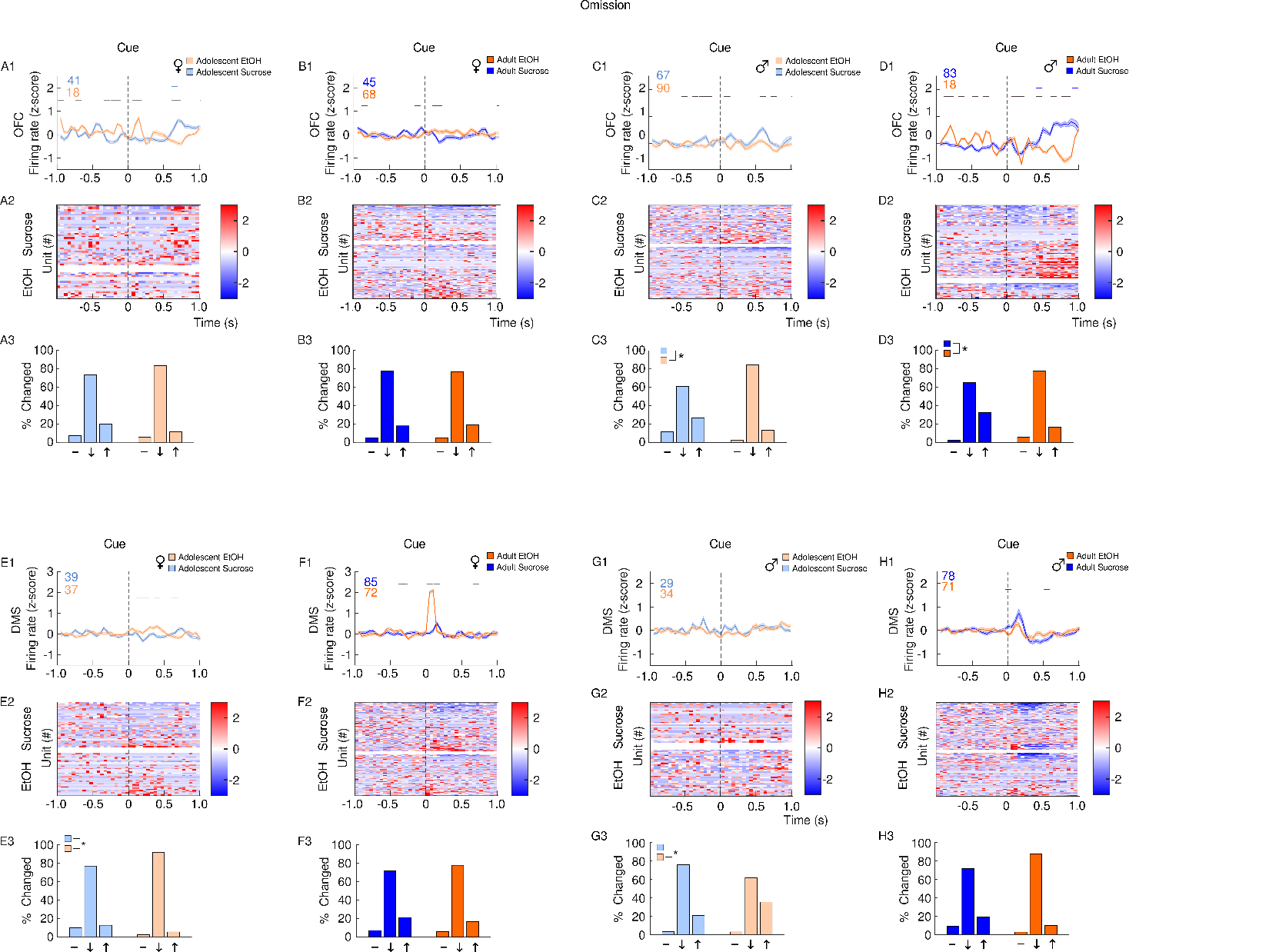
**

**Supplemental Figure 4. Single unit activity following Omissions.** Recordings were performed in the OFC (A-D) and DMS (E-H). Panels depict averaged firing rates (1), heatmaps of individual neural responses (2) and histograms (3) of the distribution of units unchanged (-), inhibited (↓) or excited (↑), 500 ms after cue presentation in male and female rats with a history of EtOH (orange) or sucrose (blue). Adolescents are plotted in the lighter shade of both colors. Events occur at time 0. In adolescent females, OFC firing rate after presentation of cues which preceded omission trials showed no apparent pattern in the overall firing rate (A1), population responses (A2) or the distribution of neural responses (A3). In adult females, OFC firing rate after presentation of cues which preceded omission trials showed no apparent pattern in the overall firing rate (B1), population responses (B2) or the distribution of neural responses (B3). In adolescent males, OFC firing rate after presentation of cues which preceded omission trials showed no apparent pattern in the overall firing rate (C1), but in the EtOH group there is an increased subpopulation of neurons that become inhibited (C2) to a greater extent than in the sucrose group (C3).In adult males, OFC firing rate after presentation of cues which preceded omission trials shows a transient increase in sucrose but not EtOH rats (D1), which is apparent in the population level response (D2). Moreover, a greater proportion of OFC neurons in the sucrose group become excited following cue presentation compared to the EtOH group (D3). In adolescent females, DMS firing rate after presentation of cues which preceded omission trials was greater in EtOH rats compared to sucrose rats (E1). While both groups had a subpopulation of excited neurons (E2), a grater proportion of DMS neurons in the EtOH group were inhibited compared to the sucrose group (E3). In adult females, DMS firing rate after presentation of cues which preceded omission trials was larger in EtOH experienced rats (F1), but both population responses (F2) and the distribution of responses were statistically similar between groups (F3). In adolescent males, DMS firing rate after presentation of cues which preceded omission trials showed no apparent pattern in the overall firing rate (G1), or population responses (A2), but sucrose animals had a larger proportion of neurons inhibited compared to EtOH rats (G3). In adult males, DMS firing rate after presentation of cues which preceded omission trials was slightly increased in sucrose rats compared to EtOH rats (H1). A subpopulation of excited and inhibited neurons was observed in both sucrose and EtOH rats (H2), but the distribution of responses was statistically similar between groups (H3). Data are presented as mean + SEM. Colored numbers reflect the number of units recorded in each group. Colored significant bars represent Tukey HSD post hoc testing (p<0.05), compared to baseline firing rate for each group (EtOH or sucrose). Black significant bars reflect permutation testing between reward groups, p<0.05. *p<0.05 group difference, Chi-squared test
